# Diagnostic Accuracy of Leucine-Rich α-2-Glycoprotein 1 as a Non-Invasive Salivary Biomarker in Pediatric Appendicitis

**DOI:** 10.3390/ijms24076043

**Published:** 2023-03-23

**Authors:** Goran Tintor, Miro Jukić, Daniela Šupe-Domić, Ana Jerončić, Zenon Pogorelić

**Affiliations:** 1 Department of Plastic Reconstructive and Aesthetic Surgery, University Hospital of Split, 21 000 Split, Croatia; gogitintor@gmail.com; 2 Department of Surgery, School of Medicine, University of Split, 21 000 Split, Croatia; mirojukic.mefst@gmail.com; 3 Department of Pediatric Surgery, University Hospital of Split, 21 000 Split, Croatia; 4 Department of Medical Laboratory Diagnostics, University Hospital of Split, 21 000 Split, Croatia; daniela.supedomic@gmail.com; 5 Department of Research in Biomedicine and Health, School of Medicine, University of Split, 21 000 Split, Croatia; ajeronci@mefst.hr

**Keywords:** acute appendicitis, children, saliva, biomarker, LRG1, leucine-rich α-2-glycoprotein 1

## Abstract

The aim of this study is to evaluate the diagnostic accuracy of leucine-rich α-2-glycoprotein 1 (LRG1) in saliva as a novel biomarker for acute appendicitis in the pediatric population. From October 2021 to June 2022, 92 children aged 5 to 17 years who presented with acute abdomen and suspected acute appendicitis were enrolled in this prospective study. The parameters documented included demographic and clinical information, as well as operative and postoperative data. Patients were divided into two groups: those with acute appendicitis who underwent laparoscopic appendectomy (*n* = 46) and those without appendicitis (*n* = 46). The total white blood cell (WBC) count, percent of neutrophils, C-reactive protein (CRP) level, and salivary LRG1 were compared between groups. A commercially available enzyme-linked immunosorbent assay (ELISA) LRG kit was used to measure the LRG levels. The median salivary LRG1 level was significantly higher in the group of children with pathohistologically confirmed acute appendicitis compared to the control group: 233.45 ng/mL (IQR 114.9, 531.2) vs. 55.95 ng/mL (IQR 51.5, 117.9), *p* < 0.001. LRG1 had an overall good receiver-operator characteristic area under the curve of 0.85 (95% CI 0.76–0.92; *p* < 0.001). The optimal LRG1 cutoff with best separation between acute appendicitis and the controls was >352.6 ng/mL (95% CI from >270.7 to >352.6). Although the specificity was 100% at this cutoff, the sensitivity for identifying appendicitis was 36%. In addition, a significant difference was found between groups in the laboratory values of all inflammatory markers tested: WBC, absolute neutrophil count, and CRP (*p* < 0.001 for all). Although LRG1 in saliva showed a good AUC parameter and significantly higher values in patients with acute appendicitis compared to the controls, its usefulness in the patient population who present at emergency departments with abdominal pain is debatable. Future studies should focus on investigating its diagnostic potential.

## 1. Introduction

Although acute appendicitis is the most common surgical emergency in children, obtaining a proper diagnosis is still difficult and often very expensive [1,2,3]. Acute appendicitis is detected in 20–30% of children who present with acute abdominal pain in a pediatric surgical emergency department [4]. In the pediatric population, the prevalence of perforated appendices ranges from 12.5 to 45%, while the risk of negative appendectomy persists at 5–25% [5]. Despite the development of numerous diagnostic tools, the diagnosis of acute appendicitis currently relies on common clinical symptoms and anamnestic evidence [1,6,7]. A wide range of factors, including atypical presentation and numerous differential diagnoses, make it difficult to establish an adequate diagnosis immediately, which increases the risk of complications [8,9,10]. In younger children, the risk of perforation is significantly higher compared to older age groups; also in addition, it was proven that the rates of perforation were significantly higher during the COVID-19 pandemic [8,10]. To improve accuracy, evaluation procedures such as computed tomography (CT) and diagnostic laparoscopy have been used; nevertheless, they are time-consuming, expensive, and intrusive (e.g., CT radiation increases the long-term cancer risk) [11,12,13,14]. A detailed history as well as a clinical examination represent the basis for making a diagnosis of acute appendicitis [15]. In addition, imaging, different scoring systems, and laboratory inflammatory markers can contribute to establishing an accurate diagnosis [1,6,11,12,13,14,15]. The majority of routinely available inflammatory biomarkers are currently insufficiently sensitive or specific to consistently confirm or exclude an appendicitis diagnosis [16,17,18]. The utilization of a screening diagnostic test, primarily one that is non-invasive, would improve the effectiveness of diagnosing and managing a pediatric population with suspected acute appendicitis.

Recent studies have suggested that leucine-rich α-2-glycoprotein 1 (LRG1) may serve as a biomarker for acute appendicitis in the pediatric population [19,20,21,22,23,24,25,26,27]. It is a 50 kD acute-phase glycoprotein, containing 312 amino acids, 66 of which are leucine in the form of the leucine-rich repeat (LRR) sequence [28,29]. LRG1 is hypothesized to be involved in neutrophil activation and chemotaxis in the inflammatory phase; however, its exact mechanism of action has yet to be determined. LRG1 has been reported to be produced and secreted by macrophages, neutrophils, liver cells, and intestinal epithelium [29,30]. Unlike C-reactive protein (CRP), which is only stimulated in the liver following interleukin 6 (IL-6) stimulation, LRG1 is also generated at the sites of lesions. In addition, LRG1 transcription is stimulated by a variety of pro-inflammatory markers, including IL-6, IL-1, IL-22, tumor necrosis factor alpha (TNF-α), and lipopolysaccharides; so, it is not dependent on a single activating factor [31,32]. Researchers have designed novel technologies and verified a variety of salivary biomarkers in recent years. The usage of saliva as a biofluid in a pediatric setting offers diverse benefits. Its acquisition is brief, simple, reasonably priced, and non-invasive. Furthermore, it can be assessed at home without medical personnel present. Consequently, the objective of our study is to analyze the applicability of salivary LRG1 in the diagnosis of acute appendicitis in the pediatric population.

## 2. Results

### 2.1. Baseline Characteristics and Clinical Data of the Patients

The demographic data of both groups of patients are summarized in Table 1. There were no statistically significant differences between the two investigated groups of patients in regard to age, gender, body weight, and height.

The intraoperative finding was positive for acute appendicitis in all of the cases from the acute appendicitis group (*n* = 46, 100%). The pathohistological analysis of the removed specimens in the acute appendicitis group is shown in Table 2.

### 2.2. LRG1 from Saliva as a Biomarker of Acute Appendicitis

In the group of children who had pathohistologically confirmed acute appendicitis, the median level of LRG1 in saliva was 233.45 ng/mL (IQR 114.9–531.2), while the median LRG1 in the control group of patients was significantly lower and was 55.95 ng/mL (IQR 28.5–117.9) (*p* < 0.001).

**Figure 1:**
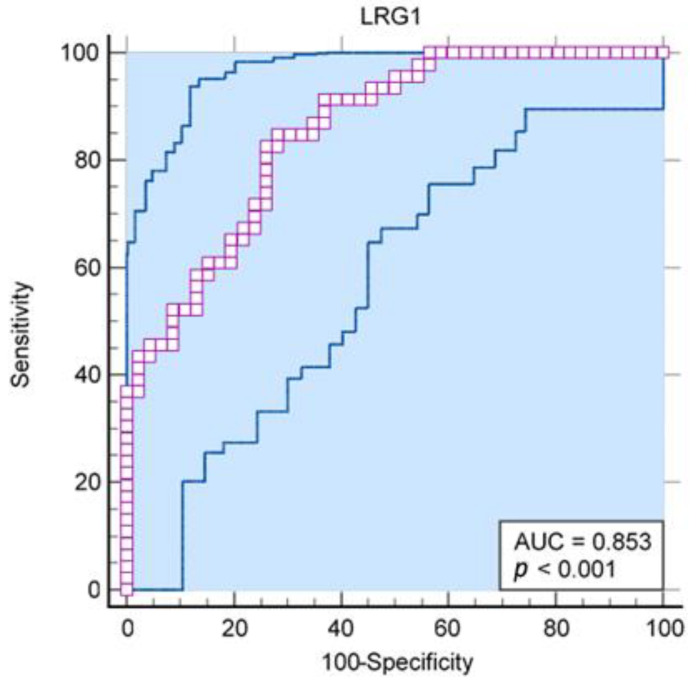
Receiver operating characteristic curve for LRG1 from human saliva as a predictor of acute appendicitis (AUC = 0.85; 95% CI 0.76–0.92; *p* < 0.001).

In addition, in our sample, the level of LRG1 in saliva enabled the excellent differentiation of acute appendicitis from the controls (AUC = 0.85; 95% CI 0.76–0.92; *p* < 0.001), while in the general population, when uncertainty is considered, one can expect from acceptable to exceptional differentiation (Figure 1).

**Figure 2:**
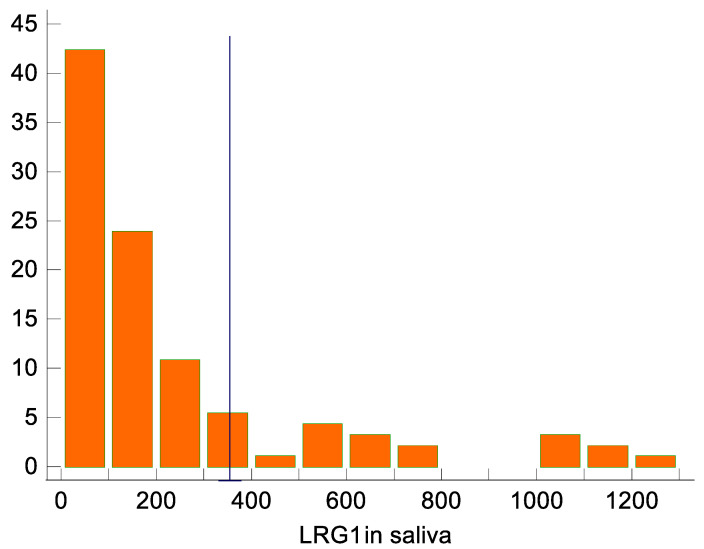
LRG1 distribution in saliva, with the marked cutoff of >352.6 ng/mL identified as optimal for distinguishing between appendicitis and non-appendicitis. Appendicitis was diagnosed in all 17 participants with >352.6 ng/mL.

The results of the Youlden index J (J = 0.57, 95% CI 0.39–0.67) and the associated criterion (criterion >103.3 ng/mL; sensitivity and specificity with respect to this criterion are 84.8% and 71.7%, respectively) are also acceptable, although they are somewhat less convincing. Regarding the optimal criterion for distinguishing appendicitis from controls, with an assumed prevalence of appendicitis of 7%, the optimal criterion for distinguishing was >352.6 ng/mL (95% CI from >270.7 ng/mL to >352.6 ng/mL; Figure 2). At this cutoff, all controls are completely below the detection threshold, but the sensitivity for identifying appendicitis is only 36%.

The diagnostic efficacy of salivary LRG1 among different pathohistological subgroups has been investigated. Salivary LRG1 levels increase with the severity of appendicitis. The AUC for phlegmonous appendicitis was 0.77 (95% CI 0.65–0.86), for gangrenous appendicitis, 0.88 (0.77–0.95), and for perforated gangrenous appendicitis, 0.99 (0.92–>0.99), although the AUC for phlegmonous appendicitis was significantly worse than for perforated gangrenous appendicitis.

### 2.3. Other Factors Associated with Acute Appendicitis

In Table 3, the clinical and laboratory data of the patients are summarized. As expected, we observed a significant increase in all investigated laboratory inflammatory markers in patients with acute appendicitis compared to those from the control group.

The diagnostic efficacy of salivary LRG1 was compared with that of common laboratory biomarkers for appendicitis: the total white blood cell count, CRP, and neutrophil count. Table 4 shows that the ability of LRG1 to predict appendicitis is comparable to that of these biomarkers. While LRG1 outperforms CRP, it performs worse than the biomarkers for the total leukocyte count or neutrophil counts.

## 3. Discussion

For many years, serum biomarkers have served as the standard and starting point for establishing a diagnosis of a disease. However, biotechnology advancements, as well as clinical requests for a more easily obtainable, non-invasive, and valuable diagnostic source of information, have resulted in the development of salivary diagnostic assays in recent years. As we enter the era of genomic technologies, pediatric patients could certainly benefit from these improvements [33,34,35]. In previous research studies, an elevation of LRG1 in the appendix as well as in the serum and urine of children with a confirmed diagnosis of acute appendicitis had been discovered [19,21,22,23,24,27]. Since its increased expression in appendicitis is considered a representation of an acute inflammatory state, it could be utilized as a prospective diagnostic instrument [36]. Accordingly, the objective of our study was to evaluate the association between the salivary biomarker LRG1 and acute appendicitis in the pediatric population. Our study demonstrated a statistically significant elevation of salivary LRG1 in children with acute appendicitis compared to the control group.

In a retrospective blinded cohort of 49 children with suspected acute appendicitis, Kentsis et al. examined the difference between urine LRG1 levels measured by ELISA from IBL International with those measured by mass spectrometry. Due to an immunoassay interference effect, the ELISA technique demonstrated inferior diagnostic performance compared to mass spectrometry, reaching AUCs of 0.80 and 0.98–0.99, respectively [21]. The strength of our study lies in the proper confirmation of acute appendicitis cases through the analysis of histopathological samples as well as in its prospective design. In terms of the study’s design, in their control group, Kakar et al. included children who had experienced a wide range of traumas, including testicular torsion, muscle tears, and fractures [27]. However, in our control group, we admitted children who presented with non-specific abdominal pain but in whom the diagnosis of acute appendicitis had been excluded by diagnostic methods in order to make a distinction between acute appendicitis and non-specific abdominal pain. Additionally, in contrast to the latter study, the LRG1 samples were not analyzed in a research laboratory but in a health care facility.

Our study corroborated the findings on the diagnostic potential of salivary LRG1 in children with acute appendicitis. The results demonstrated that LRG1 in saliva allows for an effective distinction to be made between acute appendicitis and the controls (AUC = 0.85; 95% CI 0.76–0.92; *p* < 0.001). This is comparable to the findings of a pilot study by Yap et al. who reported an AUC for salivary LRG of 0.77 (95% CI 0.60–0.93). However, when the authors used a cutoff level of 330 ng/mL, they also reported that despite achieving a specificity rate of 100%, they only saw a sensitivity rate of 35% [25]. Kharbanda et al. found that patients with appendicitis had statistically increased levels of LRG-1 in their serum and urine compared to the control group. According to their study, LRG1 could serve as a sensitive but non-specific indicator of acute appendicitis. This is attributed to the wide range of inflammatory conditions, particularly bacterial ones, that cause LRG1 up-regulation [19]. However, presumably due to the small number of participants (only 28 patients in total), the study conducted by Lontra et al. found no clinically significant difference in the serum LRG1 level between adults with and without acute appendicitis [26]. Furthermore, Salo et al. showed improved predictive values of LRG1 in urine combined with the Pediatric Appendicitis Score, achieving 95% sensitivity and 90% specificity [23,37].

Saliva contains 98% water, and numerous different significant substances make up the remaining 2%. It has been used to identify biomarkers that were traditionally acquired from blood sample analysis, including electrolytes (sodium, potassium, calcium, magnesium, hydrogen carbonates, and phosphates), cytokines (TNF-a, IL-1, IL-2, IL-6, and IL-8), various antimicrobial enzymes (a-amylase and lingual lipase), acute-phase proteins (CRP), and immunoglobulins (IgE, IgG, and IgM) [38,39,40].

As opposed to blood samples obtained by venepuncture, saliva as a diagnostic medium provides versatile advantages. The immediately apparent advantage is the non-invasive and painless nature of providing specimens, which reduces patients’ discomfort and confirms compliance, especially in the pediatric population. Furthermore, the conveniently comprehended technique for acquiring salivary samples does not rely on any professional experience. A supplementary anticoagulation sequence or the extraction of salivary proteins is not considered essential, thus enabling immediate assessment of diverse components postcollection [25].

Salivary biomarkers of neonates and children are progressively becoming particularly insightful in the preliminary prognosis and follow-up of a diverse range of conditions, such as Down syndrome, inflammatory and immune-mediated skin diseases, type 1 diabetes, and familial juvenile systemic lupus erythematosus [41,42,43]. Accurately determining the diagnosis of appendicitis is currently immensely dependent on the inflammatory biomarkers of leukocytosis and CRP taken from the blood [6]. The insufficiently high specificity and sensitivity of these markers, as well as the adverse side effects of the previously established method of blood collection, has led to the assessment of leucine-rich alpha glycoprotein 1 (LRG1) in saliva [44,45].

In addition, the potential benefit of using salivary biomarkers in patients with strongly suspected appendicitis in combination with abdominal ultrasound is that it reduces the number of unnecessary CT scans, which are associated with the risk of radiation exposure and potential harm to patients, especially children. However, there are also some limitations and challenges to consider. First, as with any diagnostic test, the sensitivity and specificity of salivary biomarkers alone may not be adequate, and false-positive or false-negative results may occur. Therefore, clinicians must carefully interpret salivary biomarker results and combine them with other clinical and laboratory findings before making a diagnosis and treatment decision. Second, the diagnostic performance of salivary biomarkers may vary depending on the stage and severity of appendicitis and the presence of confounding factors such as oral inflammation, infection, or trauma [43,46]. Therefore, careful patient selection and the standardization of salivary biomarker collection and analysis are critical to ensure reliable and accurate results. In conclusion, future studies are needed to further investigate the efficacy of salivary biomarkers as a non-invasive, radiation-free, and relatively inexpensive alternative to CT scans in this patient population.

A few limitations must be noted. This study was conceived as a single-center study, and ELISA kits from commercial sources were limited to one type. Additionally, dehydration’s impact on the LRG1 concentration levels in saliva should also be further addressed. The age limit was set at five years because it is very difficult to collect adequate samples from children under five years old.

## 4. Materials and Methods

### 4.1. Study Design and Setting

This prospective, controlled study was performed at the Department of pediatric surgery of University Hospital of Split, Croatia. From 15 October 2021 to 30 June 2022, children 5 to 17 years of age, who presented to the emergency department with acute abdominal pain and were being assessed for suspected appendicitis, were eligible for registration. The exclusion criteria were patients diagnosed with a chronic medical disease or malignancy, patients who had undergone an invasive abdominal medical procedure, and patients with a known pregnancy.

All the parents or legally authorized relatives of the patients provided their written informed consent allowing the patients to participate in the research. The study was approved by the local institutional Ethics Committee (Reference: 500-03/21-01/40; date of approval: 1 April 2021). The study was registered in the ClinicalTrials.gov registry under identifier NCT05093660.

### 4.2. Study Protocol

The following parameters were documented: complete medical history; patient’s demographic characteristics (age, sex, weight, and height); clinical signs and symptoms (duration of symptoms, pain in the right lower quadrant of the abdomen, rebound tenderness, and body temperature); information regarding surgery, pathohistological type of appendicitis (catarrhal, phlegmonous, gangrenous, or perforated); duration of surgical procedure and possible complications; and postoperative data (length of hospital stay and histological analysis). Furthermore, as part of the standardized routine care protocol for acute appendicitis, the total white blood cell (WBC) count, neutrophil percentage (Neu%), and CRP level were obtained. Seven different predictive variables were assessed through the Appendicitis Inflammatory Response (AIR) score [1]. Regarding radiological examination, ultrasonography of the abdomen and pelvis was required in all patients included in this study. For the purpose of this study, patients were divided into two groups: the acute appendicitis group (*n* = 46) consisted of patients in whom acute appendicitis was preoperatively proven by clinical examination, acute inflammatory blood markers, and abdominal ultrasound and postoperatively confirmed by pathohistology. These patients underwent laparoscopic appendectomy. Three-port access laparoscopic appendectomy was performed in all subjects in the first group as described in our previously published study [47]. The non-appendicitis group (*n* = 46) consisted of patients who presented with non-specific abdominal pain and in whom acute appendicitis could be excluded by diagnostic measures. Patients in this group were hospitalized for observation because of abdominal pain.

### 4.3. Blood Collection and Preparation

Blood was drawn from the patient’s arm veins with a butterfly needle and placed in a vial containing a clot activator and a vial containing the anticoagulant tripotassium ethylenediaminetetraacetic acid (K3 EDTA). Immediately after collection, the vials of blood were taken to the central hospital laboratory, where one vial containing the clot activator was centrifuged. The serum from the vial with the clot activator was used to measure the CRP levels. Blood collected in a vial containing K3 EDTA was used to analyze the WBC count and total neutrophils. The WBC and total neutrophil count analysis was performed in a routine laboratory on a hematology blood analyzer (Advia 2120, Bayer, Germany). A CRP assay was performed by the immunoturbidimetric method using the Cobas C702 chemistry analyzer (Roche, Rotkreuz, Switzerland).

### 4.4. Saliva LRG1 Collection

Saliva samples were extracted from participants in this study at the time of their admission using the SalivaBio Children’s Swab. Prior to saliva sampling, patients were instructed to wash their mouth with water to remove particles of food and to wait for a minimum of 5 min. Subsequently, one end of the swab stick was placed in their mouth for approximately two minutes. The swab stick was then instantly relocated from the patient’s mouth into a storage tube and placed in an ice box. Within 30 min, the swab stick was processed in the laboratory. For the purpose of extracting the patient’s saliva, the samples were centrifuged at 2500× *g* for 10 min at 4 °C. Before performing the final analysis, the prepared samples were maintained at −80 °C.

### 4.5. Saliva LRG1 Analysis

Following the manufacturer’s instructions, a commercially available enzyme-linked immunosorbent assay (ELISA) kit (IBL International, Takara, Japan) was utilized to conduct a quantitative determination of human LRG1 in the saliva. This kit contains two distinct types of highly specific antibodies in a solid phase sandwich ELISA. The coloring agent is tetra methyl benzidine (TMB). The quantity of human LRG1 has a direct connection to the color intensity. With the provided dilution buffer, saliva samples were diluted in a ratio of 1:10.

### 4.6. Final Diagnosis of Patients

The intraoperative findings were used to determine the diagnosis of acute appendicitis. Histopathology analysis was finished 2–3 weeks after surgery and further confirmed the diagnosis determined previously in depth. The exclusion of acute appendicitis was established after at least 24 h of observation in the hospital setting, where surgical procedure was not required. The final, histologically verified diagnosis was kept confidential from the scientists who processed the LRG1 level in the saliva.

### 4.7. Sample Size Calculation

Assuming a statistical power of 80% and a significance level of 0.05, the area under the curve (AUC) for the null hypothesis of 0.50 and 0.69 for the alternative hypothesis, and a ratio of negative to positive cases of appendicitis of 2, a minimum of 82 subjects (41 per group) must be drawn from the consecutive sampling of children presenting to a pediatric emergency department with possible appendicitis. Overall, we aimed for the minimum number of participants of 46 subjects per group to ensure sufficient statistical power for possible dropouts due to negative appendectomies or inferences about the variables studied.

### 4.8. Statistical Analysis

For the statistical analysis, SPSS 24.0 software (IBM Corp, Armonk, NY, USA) was used. The distributions of the qualitative data were described with absolute and relative frequencies, whereas the distributions of the quantitative data were described with mean and standard deviation or median and interquartile range, depending on the normality of the data. We used the D’Agostino-Pearson test to determine the normality of the data, and then used the independent t-test or its non-parametric alternative, the Mann–Whitney test, to infer differences between patient groups. To measure the usefulness of a diagnostic test based on the LRG1 data, we performed a receiver operating characteristic (ROC) curve analysis according to the methodology of deLong et al. [48] and used the AUC and Youden index J to capture the performance of the test. The optimal criterion for separating groups that considers the prevalence of disease was also calculated under the assumption taken from the literature that overall, 7% of children presenting with abdominal pain have acute appendicitis [49,50,51,52].

## 5. Conclusions

Although LRG1 in saliva showed a good AUC parameter and significantly higher values in patients with acute appendicitis compared to the controls, its usefulness in a patient population who present at the hospital emergency department with abdominal pain is debatable. To promote the attention of researchers on salivary biomarkers for other pediatric and neonatal diseases in addition to appendicitis, well-designed prospective diagnostic comparative effectiveness studies are crucial.

## Figures and Tables

**Table 1:** Demographic data of the patients.

Variables	Acute Appendicitis (*n* = 46)		Non-Appendicitis (*n* = 46)		*p*
	Mean	SD	Mean	SD	
Age (years)	11.4	3.3	11.8	3.0	0.602 *
Body weight (kg)	47.4	17.1	48.0	16.1	0.861 *
Body height (cm)	154.3	19.6	154.9	16.3	0.876 *
Gender	*n*	%	*n*	%	
Male	33	71.7	13	28.3	0.130 ^‡^
Female	26	56.5	20	43.5	

SD—standard deviation; * independent samples *t*-test; ‡ Chi-square test.

**Table 2:** Pathohistological analysis of removed specimens in the acute appendicitis group.

Pathohistological Findings	*n* (%)
Phlegmonous appendicitis	20 (43.5)
Gangrenous appendicitis	16 (34.8)
Perforated gangrenous appendicitis	10 (21.7)

**Table 3:** Clinical and laboratory data of the patients.

Variables	Acute Appendicitis (*n* = 46)		Non-Appendicitis (*n* = 46)		p *
	Median	IQR	Median	IQR	
Duration of symptoms (h)	25	(18, 36)	32.5	(24, 50)	0.031
AIR score	9	(7, 10)	3	(3, 4)	<0.001
Body temperature (°C)	37.3	(36.9, 37.6)	36.8	(36.6, 36.9)	<0.001
WBC (×10^9^/L)	14.6	(12.7, 18.7)	7.0	(5.4, 9.0)	<0.001
CRP (mg/dL)	16.3	(6.9, 50.4)	2.2	(2, 2)	<0.001
Neutrophil count (%)	84.6	(79.5, 89.0)	59.5	(51.5, 68.6)	<0.001
LRG1 in saliva (ng/dL)	233.45	(114.9, 531.2)	55.95	(51.5, 117.9)	<0.001
Duration of surgery (min)	21	(18, 30)	-	-	-
Length of hospital stay (days)	2	(1, 3)	2	(2, 2)	0.856

IQR—interquartile range; AIR—Appendicitis Inflammatory Response; WBCs—white blood cells; CRP—C-reactive protein; LRG1—leucine-rich α-2 glycoprotein 1; * Mann–Whitney test.

**Table 4:** The diagnostic potential of commonly used laboratory markers for acute appendicitis.

Biomarker	AUC (95% CI)	Youlden Index J		Sensitivity in Population [%] *
		J	Sensitivity and specificity [%]	
WBC (×10^9^/L)	0.94 ** (0.86–0.98)	0.83 (0.69–0.91)	95.7, 87.0	78.3
CRP (mg/dL)	0.76 ** (0.65–0.84)	0.46 (0.27–0.59)	78.3, 67.4	10.9
Neutrophil count (%)	0.95 ** (0.88–0.98)	0.78 (0.63–0.87)	84.8, 93.5	65.2

AUC—area under the curve; WBCs—white blood cells; CRP—C-reactive protein. * considering disease prevalence of 7% in target population; ** significant at 0.001 level.

## Data Availability

The data presented in this study are available upon request of the respective author. Due to the protection of personal data, the data are not publicly available.
